# Preterm Birth Impacts the Timing and Excursion of Oropharyngeal Structures during Infant Feeding

**DOI:** 10.1093/iob/obaa028

**Published:** 2020-08-27

**Authors:** C E Edmonds, E A Catchpole, F D H Gould, L E Bond, B M Stricklen, R Z German, C J Mayerl

**Affiliations:** 1 Department of Anatomy and Neurobiology, Northeast Ohio Medical University (NEOMED), Rootstown, OH 44272, USA; 2 Department of Cell Biology and Neuroscience, Rowan School of Osteopathic Medicine, Stratford, NJ 08854, USA

## Abstract

Swallowing in mammals requires the precise coordination of multiple oropharyngeal structures, including the palatopharyngeal arch. During a typical swallow, the activity of the palatopharyngeus muscle produces pharyngeal shortening to assist in producing pressure required to swallow and may initiate epiglottal flipping to protect the airway. Most research on the role of the palatopharyngeal arch in swallowing has used pharyngeal manometry, which measures the relative pressures in the oropharynx, but does not quantify the movements of the structures involved in swallowing. In this study, we assessed palatopharyngeal arch and soft palate function by comparing their movements in a healthy population to a pathophysiological population longitudinally through infancy (term versus preterm pigs). In doing so, we test the impact of birth status, postnatal maturation, and their interaction on swallowing. We tracked the three-dimensional (3D) movements of radiopaque beads implanted into relevant anatomical structures and recorded feeding via biplanar high-speed videofluoroscopy. We then calculated the total 3D excursion of the arch and soft palate, the orientation of arch movement, and the timing of maximal arch constriction during each swallow. Soft palate excursion was greater in term infants at both 7 and 17 days postnatal, whereas arch excursion was largely unaffected by birth status. Maximal arch constriction occurred much earlier in preterm pigs relative to term pigs, a result that was consistent across age. There was no effect of postnatal age on arch or soft palate excursion. Preterm and term infants differed in their orientation of arch movement, which most likely reflects both differences in anatomy and differences in feeding posture. Our results suggest that the timing and coordination of oropharyngeal movements may be more important to feeding performance than the movements of isolated structures, and that differences in the neural control of swallowing and its maturation in preterm and term infants may explain preterm swallowing deficits.

## Introduction

The mammalian swallow is a complex physiological process that requires the precise coordination of several oropharyngeal structures, including the tongue, soft palate, hyoid bone, thyroid cartilage, epiglottis, and palatopharyngeal arch. In turn, over 25 paired muscles innervated by multiple cranial nerves power these structures (Miller 1986; Jean 2001; German et al. 2009; Thexton et al. 2012). However, understanding the kinematics and coordination of the different components involved in swallowing can be challenging. First, swallowing is hidden, and can only be observed using videofluoroscopy. Second, there are no synovial joints involved in swallowing, and the primary structures are either suspended in a muscular sling, like the hyoid bone (German et al. 2011; Pearson et al. 2012), or are composed solely of soft tissues, like the tongue (Napadow et al. 1999; Gilbert et al. 2007; Kieser et al. 2014). Third, most previous research on swallow kinematics has been limited to two-dimensional (2D) analyses, with some notable exceptions (Orsbon et al. 2018). Because of these constraints, we lack insight into how the different pieces of anatomy driving swallowing move and are coordinated with each other. 

One structure that plays an important role in the swallow is the palatopharyngeal arch. The palatopharyngeal arch is formed by mucosal coverings of the paired palatopharyngeus muscles which contract to assist in pharyngeal shortening and constriction during the swallow. Constriction of the palatopharyngeal arch, along with activity of the tensor veli palatini, levator veli palatini, palatoglossus, and uvula, results in movement of the soft palate to protect the nasopharynx and provide pressure to move the bolus (Podvinec 1952; Hennessy and Goldenberg 2016; Rathod et al., 2018). Additionally, arch constriction may cause the epiglottis to flip and cover the airway (Crompton et al. 2008). However, the relative timing and movement of the palatopharyngeal arch remains largely unexplored and has been restricted to 2D analyses, despite its 3D nature. Furthermore, because of radiation concerns, past studies evaluating pharyngeal constriction have often been limited to pharyngeal manometry (Rommel et al. 2011; Prabhakar et al. 2019). Manometry, which measures the relative pressures in the pharynx during the swallow (Hurwitz et al. 1975; Castell 1993), cannot quantify the 3D excursion of oropharyngeal structures, nor identify how changes in the movement of these structures contribute to pressure differences.

One common tool for understanding the function of a structure is to compare how it behaves in healthy populations to its behavior in compromised populations. For example, the role of the interior branch of the Superior Laryngeal Nerve (iSLN) as the trigger for initiating the swallow reflex can be demonstrated by comparing swallow properties in intact versus lesioned populations (Ding et al. 2013, 2015; Lammers et al. 2020). Similarly, the role of the palatopharyngeal arch in swallowing might be better understood by studying how it functions in populations that are compromised. Preterm infants are widely considered to be a neurologically compromised population (Barlow 2009), and up to 80% of prematurely born infants experience feeding difficulties (Bryant-Waugh et al. 2010), that are thought to result primarily from decreased uterine neural maturation resulting in challenges associated primarily with coordinating multiple behaviors needed for safe and effective feeding (Amaizu et al. 2007; Delaney and Arvedson 2008; Mayerl et al. 2019). These challenges are pervasive, can last through infancy and beyond (Burklow et al. 2002; Dodrill et al. 2004; Lau 2006), and include decreased abilities to acquire and transport food (Mayerl et al. 2020a; Capilouto et al. 2014), as well as to coordinate different behaviors associated with the swallow (Rommel et al. 2011; Catchpole et al. 2020; Mayerl et al. 2019, 2020b), and to coordinate the swallow with respiration (Gewolb and Vice 2006; Lau et al. 2007; Mayerl et al. 2019). Preterm infants also exhibit decreased pharyngeal contractility, and thus represent an ideal population for studying pharyngeal dysfunction (Prabhakar et al. 2019).

**Fig. 1:**
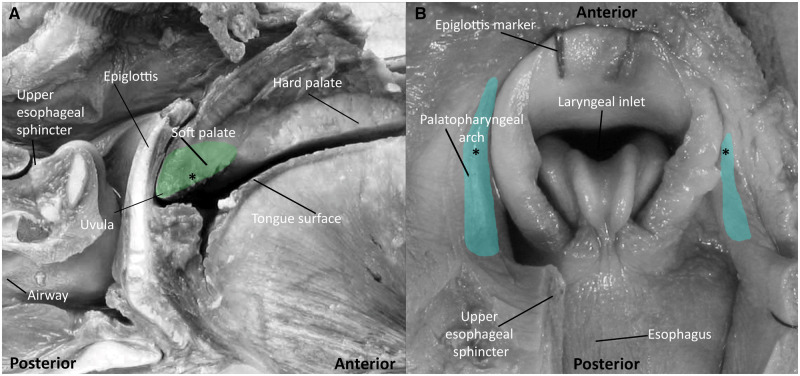
The oropharyngeal anatomy of the infant pig. (**A**) Midsagittal section, with the soft palate highlighted in green. (**B**) A dorsal view of the laryngeal inlet, with the palatopharyngeal arch highlighted in blue. Symbol “*” indicates the placement of radiopaque markers in soft tissue structures for tracking *in vivo* kinematics. Figure modified with permission from Crompton et al. (2008), original photograph by C. Musinsky.

In this study, we studied palatopharyngeal arch movement and its relative timing during infant bottle feeding in order to assess the role of the arch in both normal and pathophysiological swallowing. We used a validated infant pig model (Thexton et al. 2004, 2012; German et al. 2017) to evaluate the effects of preterm birth, postnatal maturation, and their interaction on the behavior of the palatopharyngeal arch while swallowing milk. Infants represent an excellent model for understanding swallowing in mammals because the swallow is not impacted by variation in bolus properties or oral processing depending on different food types, as it is in adults (Hiiemae and Crompton 1985). We recorded 3D movements of radiopaque tantalum markers placed in the palatopharyngeal arch and the soft palate of term and preterm infant pigs (Fig. 1). Using high-speed biplanar videofluoroscopy, we measured total arch and soft palate excursion, timing of maximal arch constriction, and orientation of arch movement with the following predictions.

Because preterm infants have decreased pharyngeal contractility (Prabhakar et al. 2019), we predicted that the excursion of the soft palate and palatopharyngeal arch of preterm pigs would be less than their term counterparts throughout infancy. We also predicted that preterm pigs’ arches would reach the point of maximal constriction earlier in the swallow than those of term pigs, as they swallow smaller boluses of milk than term pigs (Mayerl et al. 2020a), and may have narrower pharynxes, which suggests that the palatopharyngeal arch would reach its point of maximum constriction earlier. Finally, we predicted that with postnatal maturation, palatopharyngeal arch orientation would become more anteroposterior (AP), and less mediolateral (ML), as infants possess an anteroposteriorly compressed oropharyngeal anatomy that elongates with age. These data represent a first step toward understanding the role of the palatopharyngeal arch during swallowing and provide evidence that the timing and coordination of oropharyngeal structures driving swallowing performance may be just as important as understanding the role of the overall movement of those structures.

## Materials and methods

### Animal delivery and care

Infant pigs utilized in this experiment were acquired from a Yorkshire/Landrace cross pregnant sow (Shoup Farms, Wooster, OH) via Cesarean section at 115 days gestation (full term) or 108 days gestation (preterm; 30–32 weeks gestation human equivalent [Eiby et al. 2013]). Cesarean section removes the effect of vaginal birth on term infant pigs, ensuring differences in performance are not due to vaginal delivery. Details on the C-section can be found in Ballester et al. (2018), but in short, the sow was sedated with Telazol (4–10 mL intramuscular) and anesthesia was administered and maintained with isoflurane (2–5%). The surgical site was prepared by shaving the sow’s right mid-abdominal flank and scrubbing it with betadine and 70% isopropyl alcohol. The incision site was treated with lidocaine (15 mL subcutaneous) and sterile drapes were placed over the sow. To remove the infant pigs, an incision was made to expose the uterus, and then the uterus was cut to extract the infant pigs one at a time. After it was confirmed that all infant pigs were delivered, the mother sow was euthanized.

As each infant pig was extracted, the umbilical cord was clamped and cut, and infant pigs were individually wrapped in warm sterile towels. Fluid was removed from the airway first with gravity and then by suction, if necessary. If any of the infant pigs showed symptoms of respiratory depression, they received doxapram sublingually (0.1–0.2 mL) to reestablish respiration. Weak breathers were placed adjacent to strong breathers to promote improved respiration.

Once breathing became relatively strong and stable, infant pigs were transported to a standard infant incubator (Dräger Medical Isolette Infant Incubator C2000, Telford, PA) which maintained a temperature of 30°C. Within 2 h of delivery, pigs were fed colostrum from a syringe (CL Sow Replacer Cuprem Inc., Kennesaw, NB). Over the course of the first 24 h of birth, infant pigs were transitioned to formula fed from infant bottles and nipple (Solustart Pig Milk Replacement; Land O’ Lakes; Arden Mills, MN; and NASCO Farm & Ranch, Fort Atkinson, WI). During the first week of life, pigs were placed under 24-h surveillance to ensure that all animals were thriving. For the remainder of the experiment, animal care was consistent with validated standard care for neonatal pigs (Gould et al. 2016; Ballester et al. 2018). All procedures carried out through the duration of this experiment were in compliance with NEOMED IACUC protocol No. 17-04-071.

### Marker implantation and RLN lesion surgery

Animals utilized in this study were part of a larger study on determining the effects of recurrent laryngeal nerve (RLN) lesion and preterm birth on infant feeding performance (Mayerl et al. 2020a, 2020b). Shortly after birth, each animal was randomly assigned to one of two groups: the control group or the treatment (lesion) group. Only control individuals were used in these analyses. All pigs had 0.8 mm radiopaque tantalum beads implanted intraorally in the tongue, hard palate, soft palate, palatopharyngeal arch, and nose while under anesthesia (2–5% isoflurane) when they were 5–6 days old. A microvascular clip was placed on the epiglottal tip to track movements of the epiglottis during feeding.

In a separate procedure, 5–6 days after birth, we performed a sterile surgery on all pigs to suture radio-opaque beads in the tissues surrounding the hyoid and thyroid. The hyoid marker was placed by following the sternohyoid muscles to their anterior insertion at the hyoid and suturing a bead between the two bellies at that location. The thyroid bead was sutured into the fascia over the thyroid eminence just anterior to the insertion of the sternothyroid muscle. During this surgery, the control animals’ right RLN was identified.

### Feeding and data collection

We collected data when the pigs were 7 days old (equivalent to 1–2 months human age), and when they were 17 days old (equivalent to 6–9 months human age). Seven days after birth is the earliest time point that data can be collected, as prior to that time point infant pigs cannot maintain a stable body temperature needed for transport from the animal facility to data collection facilities. At 17 days of age, premolars have erupted, and pigs begin to show an interest in solid food, marking the beginning of the transition to solid food and one of the last time points at which pigs are feeding exclusively on milk (Bond et al. 2020). By the time infant pigs reach 7 days postnatal, mass differences between term and preterm infants used in this experiment are negligible and do not require an adjustment for body size (Mayerl et al. 2020b). For both age groups, pigs were trained to drink formula mixed with barium (E-Z Paque Barium Sulfate, EZ EM Inc., NY) from a bottle. Training occurred while animals stood in a transparent, radiolucent box constructed specifically for this application.

While the pigs were feeding, we collected biplanar fluoroscopic data using two fluoroscopes (GE9400 C-Arm, 65 kV, 5 mA) outfitted with digital high-speed cameras (XC1M digital camera; XCitex, Cambridge, MA) at 100 frames/s, positioned in dorsoventral (DV) and lateral orientations (Supplementary Movie S1). The radiolucent box was placed in front of the fluoroscopes, and pigs were allowed to suckle until satiation. Data collection procedures followed standard X-Ray Reconstruction of Moving Morphology (XROMM, Brainerd et al. 2010) protocols.

### Processing kinematic data

Detailed information on marker tracking and data processing can be found in Mayerl et al. (2020b). In order to ensure that all swallows examined were representative of typical infant feeding, we did not consider the first 5 s after the animal latched onto the nipple, as swallows within this time period occur at a much faster rate than the remainder of the feeding sequence (Gierbolini-Norat et al. 2014). Following this initial burst of feeding, 20 swallows were identified per pig, per age for tracking and analysis. Swallow onset was defined as the time that the bolus was condensed completely in the supraglottic space (Ballester et al. 2018; Mayerl et al. 2019). Swallow conclusion was identified as the frame where the epiglottis returned to its original resting position.

Kinematic data were processed using XMALab (Knörlein et al. 2016) to acquire 3D coordinates of each marker. Data were filtered at 10 Hz with a low-pass filter to reduce noise and all data were checked for appropriate filtering in XMALab prior to exportation. Markers placed in the nose and hard palate had intermarker distance standard deviations of less than 0.03 mm and were assigned to a rigid body. Translations of the hard palate-nose rigid body, *XYZ* points of individual markers, and undistorted video from both views were exported to Autodesk Maya (Autodesk Inc., San Rafael, CA) for further processing. An anatomical coordinate system (ACS) was parented to the rigid body and placed at the midline on the anterior-most portion of the hard palate (Supplementary Movie S1). We chose to set our coordinate plane by defining AP movements as translation along the *x*-axis, ML movements as translation along the *y*-axis, and DV movements as translation along the *z*-axis. This enabled us to calculate *XYZ* translation for each oropharyngeal marker relative to the coordinate axis (oRel, XROMM Maya tools), which eliminated any effect of skull movement or pig location and orientation on 3D marker movement. We exported *XYZ* translations of the palatopharyngeal arch and soft palate and processed these data using a custom MATLAB code.

The MATLAB code identified the following variables: timing of maximal arch constriction during a swallow (as a percentage of swallow duration), the total excursion of the palatopharyngeal arch and the soft palate during each swallow (mm), and the 3D orientation of arch movement. We calculated 3D orientation of movement for each swallow by analyzing arch excursion in the AP, ML, and DV directions (Supplementary Table S2), and then dividing movement by total arch excursion to quantify the proportion of movement in each direction.

### Consideration of postural variation

**Fig. 2:**
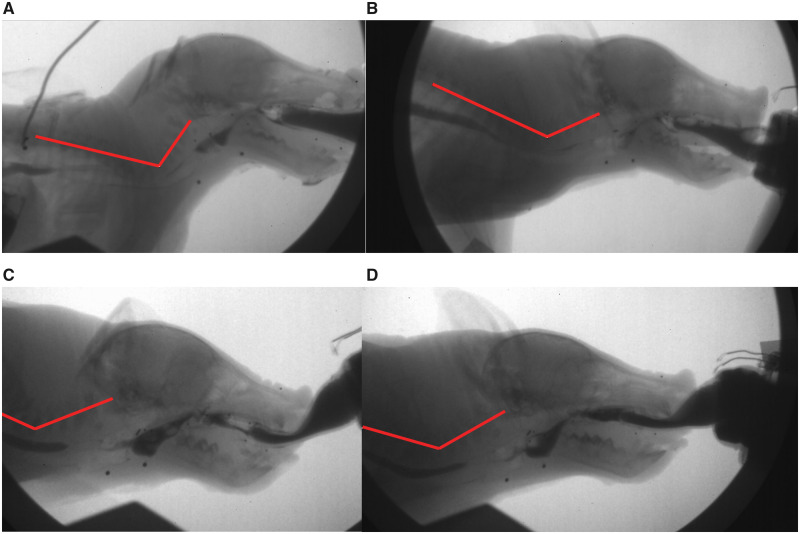
Representative head posture measurements for pigs of different birth statuses and postnatal ages. (**A**) Preterm pig, postnatal day 7. (**B**) Preterm pig, postnatal day 17. (**C**) Term pig, postnatal day 7. (**D**) Term pig, postnatal day 17.

Although the radiolucent feeding box restricted extraneous pig movement and ensured a generally consistent feeding position, there was still a potential for pigs to differ in posture while eating. Some pigs were observed eating with their neck extended, and therefore the angle formed by their head and neck was more obtuse. This was qualitatively different between preterm and term infants, especially at postnatal day 7, and could impact the relative magnitude of arch movement in the DV plane (as a pig that holds its head higher during feeding may change the orientation of its palatopharyngeal arch relative to its body). In order to determine whether differences in the orientation of arch movement were due to anatomical differences or a result of differing posture, we analyzed the angle of the cervical and trunk vertebrae of each pig at the onset of the swallow. We randomly selected five swallows for preterm pigs at each age (*N* = 25 swallows) and eight swallows for each term pig at each age (*N* = 28 swallows). We used the angle tool in ImageJ (Schneider et al. 2012) to identify (1) the first cervical vertebra (C1), the sixth cervical vertebra (C6), and the thoracic vertebral line (Fig. 2).

### Statistical analysis

All statistical analyses were performed in R (v. 3.6.3, http://www.r-project.org). We used linear mixed-effect models with birth status, postnatal age, and their interaction as fixed effects, while individual pig was treated as a random effect [Response ∼ Treatment × Age + (1|Individual); lmer4 (Bates et al. 2015)]. This analysis allowed us to evaluate the impact of postnatal maturation and birth status and their interaction on the excursion of the soft palate and palatopharyngeal arch, timing of arch constriction, orientation of arch excursion, and the angle of the head and neck during feeding. *P*-values were obtained using likelihood ratio tests of the full model with the effect in question against the model without the effect in question. We used planned contrast analyses to find specific effects of birth status and postnatal age within groups (R package emmeans). Data used in statistical analyses are available upon request.

## Results

### 3D palatopharyngeal arch excursion

**Fig. 3:**
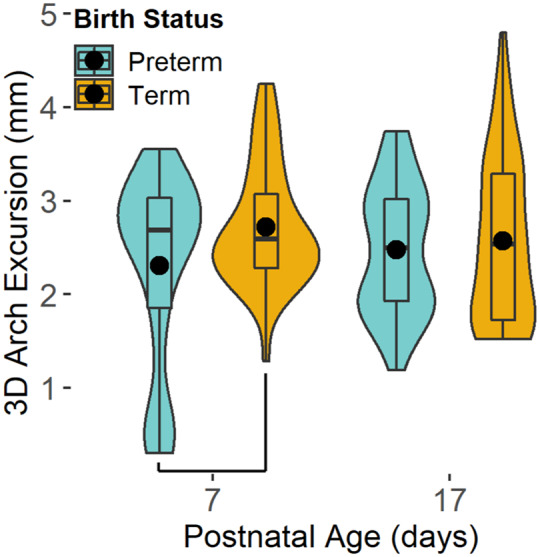
Palatopharyngeal arch excursion in preterm (blue) and term (yellow) pigs at day 7 (left) and 17 (right). At 7 days postnatal, there is a significant effect of birth status on arch excursion. Black circles represent means; box and whisker plots show median and interquartile range; width of each plot indicates the frequency distribution of the data along the *y*-axis; and brackets connecting plots show significant differences identified by planned contrast analyses (*P* < 0.05).

A significant interaction existed between postnatal age and birth status on 3D palatopharyngeal arch excursion (Fig. 3 and Supplementary Table S1). At 7 days postnatal, arch excursion was greater in term infants than preterm infants (term mean = 2.72 ± 0.10 mm, preterm mean = 2.31 ± 0.13 mm, *t*-ratio = 2.52, *P* = 0.013). However, the same relationship was not observed at 17 days postnatal, where there was no effect of birth status between term and preterm arch excursion (term mean = 2.57 ± 0.12 mm, preterm mean = 2.47 ± 0.10 mm, *t*-ratio = 0.57, *P* = 0.568). There was no effect of postnatal age on arch excursion, which was similar at 7 and 17 days postnatal in term infants (*t*-ratio = −0.87, *P* = 0.384) and preterm infants (*t*-ratio = 1.02, *P* = 0.311, Supplementary Table S1).

### 3D soft palate excursion

**Fig. 4:**
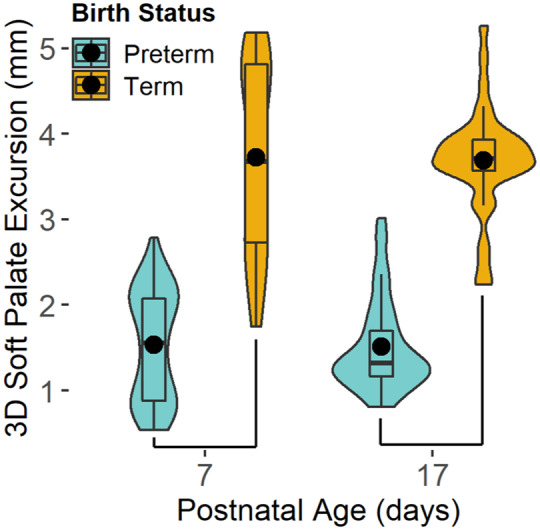
Soft palate excursion in preterm (blue) and term (yellow) pigs at days 7 (left) and 17 (right). At both 7 and 17 days postnatal, there is a significant effect of birth status on soft palate excursion. Black circles represent means; box and whisker plots show median and interquartile range; width of each plot indicates the frequency distribution of the data along the *y*-axis; and brackets connecting plots show significant differences identified by planned contrast analyses (*P* < 0.05).

There was a significant interaction of postnatal age and birth status on 3D soft palate excursion during the swallow (Fig. 4 and Supplementary Table S1). At 7 days postnatal, soft palate excursion was greater for term pigs than preterm pigs (term mean = 3.72 ± 0.17 mm, preterm mean = 1.53 ± 0.08 mm, *t*-ratio = 14.99, *P* < 0.0001). A similar relationship existed at postnatal day 17, with higher soft palate excursion in term pigs (term mean = 3.69 ± 0.10 mm, preterm mean = 1.51 ± 0.08 mm, *t*-ratio = 13.02, *P* < 0.0001). There was no effect of postnatal age on soft palate excursion, which was similar at 7 and 17 days postnatal in term infants (*t*-ratio = −0.19, *P* = 0.854) and preterm infants (*t*-ratio = −0.17, *P* = 0.867).

### Timing of maximal arch constriction

**Fig. 5:**
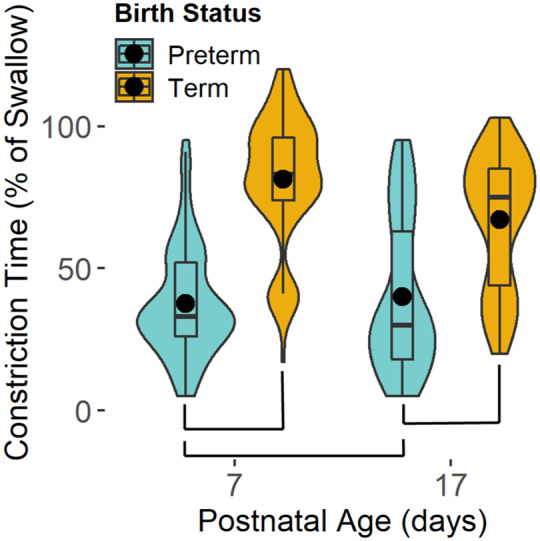
Timing of maximal palatopharyngeal arch constriction (as a percentage of the swallow) in preterm (blue) and term (yellow) pigs at days 7 (left) and 17 (right). At both 7 and 17 days postnatal, there is a significant effect of birth status on timing of arch constriction. Black circles represent means; box and whisker plots show median and interquartile range; width of each plot indicates the frequency distribution of the data along the *y*-axis; and brackets connecting plots show significant differences identified by planned contrast analyses (*P* < 0.05).

There was a significant effect of birth status on the timing of maximal palatopharyngeal arch constriction (Fig. 5 and Supplementary Table S1). At postnatal day 7, term pigs experienced maximal arch constriction much later in the swallow cycle than preterm pigs (term mean = 83.86 ± 1.73%, preterm mean = 35.38 ± 2.72%, *t*-ratio = 16.35, *P* < 0.0001). The same effect of birth status existed at postnatal day 17 (term mean = 81.15 ± 1.53%, preterm mean = 23.14 ± 1.33%, *t*-ratio = 19.12, *P* < 0.0001). There was also a significant interaction between birth status and postnatal age. At postnatal day 17, preterm pigs experienced maximal arch constriction earlier in the swallow than at postnatal day 7 (*t*-ratio = −4.19, *P* < 0.0001).

### Orientation of arch movement and postural analysis

Regardless of postnatal age or birth status, the palatopharyngeal arch consistently moved in three dimensions during the swallow. On postnatal day 7, preterm pigs had palatopharyngeal arch movements in a more ML orientation than term pigs, whereas term pigs had a greater proportion of movement in both AP and DV directions (Table 1 and Supplementary Table S2). On postnatal day 17, palatopharyngeal arch movements in preterm pigs were proportionally greater in the DV direction and smaller in the AP direction (Table 1 and Supplementary Table S2). Older term pigs had less DV and more ML movement than younger pigs, whereas the opposite was true in preterm pigs (Table 1 and Supplementary Table S2).

**Table 1:** Mean values for palatopharyngeal arch excursion, soft palate excursion, timing of maximal palatopharyngeal arch constriction during the swallow, proportion of total arch excursion in the anteroposterior (AP) direction, proportion of total arch excursion in the mediolateral (ML) direction, proportion of total arch excursion in the dorsoventral (DV) direction, and head angle during feeding

	Preterm, day 7	Preterm, day 17	Term, day 7	Term, day 17
Mean arch excursion (mm)	2.31 ± 0.13	2.47 ± 0.10	2.72 ± 0.10	2.57 ± 0.12
Mean soft palate excursion (mm)	1.53 ± 0.08	1.51 ± 0.08	3.72 ± 0.17	3.69 ± 0.10
Mean arch constriction timing (% of swallow)	35.38 ± 2.72	23.136 ± 1.33	83.86 ± 1.73	81.15 ± 1.53
Mean AP arch movement (%)	34.44 ± 2.63	38.05 ± 1.72	47.50 ± 2.52	47.91 ± 1.36
Mean ML arch movement (%)	33.17 ± 3.25	16.18 ± 1.52	14.84 ± 1.06	20.11 ± 0.71
Mean DV arch movement (%)	32.36 ± 1.09	45.75 ± 1.78	37.64 ± 1.75	31.96 ± 1.06
Mean head angle (°)	116.32 ± 1.89	142.62 ± 2.42	129.08 ± 1.85	134.08 ± 1.26

A significant effect of the interaction between birth status and postnatal age on head angle existed (Supplementary Table S3), where term pigs on postnatal day 7 had head angles approximately 13° more oblique than preterm pigs (term mean = 129.08 ± 1.85°, preterm mean = 116.32 ± 1.89°, *t*-ratio = 4.84, *P* < 0.0001). By postnatal day 17, these differences were less, with preterm pigs exhibiting more oblique head angles than term pigs (term mean = 134.08 ± 1.26°, preterm mean = 142.62 ± 2.42°, *t*-ratio = −3.24, *P* = 0.002). There was a significant effect of postnatal age on head posture during the feeding sequence, with younger pigs exhibiting a more upright posture during feeding, regardless of gestational age at birth (Fig. 2 and Supplementary Table S3).

## Discussion

The palatopharyngeal arch has been hypothesized to contribute to swallow safety and efficiency in a number of ways, including moving the soft palate, constricting the pharynx, and flipping the epiglottis (Podvinec 1952; Crompton et al. 2008; Hennessy and Goldenberg 2016; Rathod et al. 2018). Our findings provide some insight into the role of the palatopharyngeal arch during the swallow via 3D tracking of arch kinematics. Contrary to our expectation, arch excursion did not differ between term and preterm infants. However, preterm birth resulted in decreased soft palate excursion at both 7 and 17 days postnatal, as well as consistently earlier maximal arch constriction. We also found substantive changes in the orientation of arch movement and feeding posture in preterm, but not term infants, partially supporting our prediction. These results imply that oropharyngeal movements in term and preterm infants are distinct, and that these infants mature postnatally in different ways.

### Preterm birth affects coordination of soft palate muscles

Our results suggest that compared with term infants, preterm infants experience slightly decreased palatopharyngeal arch excursion at 7, but not 17 days postnatal, although these statistical differences are small, and likely not biologically relevant. There are multiple possibilities for a lack of differences in arch excursion between groups. One possibility is that the amount of interindividual variation in bead placement may overwhelm any signal present in arch excursion. The palatopharyngeal arch is a small structure running underneath a layer of mucosa, and slight differences in the placement of markers might introduce variation in the movement of the arches. Future work may want to place multiple beads within the muscle in order to measure muscle strain patterns using fluoromicrometry (Camp et al. 2016). On the other hand, similar arch excursion between term and preterm infants at both ages could be reflective of biology: the palatopharyngeal arch may constrict only a limited amount, which does not change postnatally. If the arch were functioning primarily to cause the epiglottis to invert, this could certainly be the case (Crompton et al. 2008).

Soft palate excursion may be a more accurate method of quantifying oropharyngeal function. The soft palate is a larger, more conspicuous structure, and marker placement is very consistent and straightforward. Because soft palate excursion relies on the concurrent activity of the palatopharyngeus and four other muscles, any change in the contraction of the palatopharyngeus should alter soft palate movements. Furthermore, the precise coordination of all five soft palate muscles is essential to proper function (Podvinec 1952; Hennessy and Goldenberg 2016; Rathod et al. 2018). Any disturbance to the activity of these muscles or the nerves that innervate them could cause disorganization of the entire system, leading to observable deficits in soft palate function.

Decreased soft palate excursion but not palatopharyngeal arch excursion in our data may be a result of the neurological deficits associated with preterm birth (Barlow 2009; Barlow et al. 2014). Rather than impacting the excursion of any one structure or movement, preterm birth appears to have resulted in decreased excursion of a structure that requires synchronous activation of multiple muscles. This suggests that preterm infants may not exhibit as much coordination among the muscles of the soft palate as their term counterparts do. This is consistent with previous studies which have identified that preterm infants have trouble coordinating among the behaviors within a swallow and feeding sequence, more so than they show any deficits within individual behaviors (Gewolb and Vice 2006; Lau et al. 2007; Mayerl et al. 2019; Catchpole et al. 2020; Mayerl et al. 2020b). Insufficient elevation and depression of the soft palate will also interfere with the generation of positive pressure that propels the bolus into the pharynx, which could explain the formation of smaller boluses in preterm infant pigs (McConnel 1988; Mayerl et al. 2020c). Additionally, if the soft palate is not sufficiently mobile, it will not seal off the nasopharynx, which could contribute to the poor airway protection that is often observed in preterm infants (Crompton et al. 1997).

### Orientation of arch movement changes as infants mature

We also observed changes in palatopharyngeal arch orientation due to both preterm birth, and postnatal maturation. These changes are likely a result of both how the head was positioned, as well as the anatomy of the oropharynx. While term infants had similar head orientations throughout nursing, younger preterm infants held their heads in a much more upright position than older preterm infants, the latter of which were similar to term infants. This change in posture could have resulted in the increase in DV movement postnatally in these infants. In contrast, term infants did not change their head orientation substantively postnatally, and their changes in arch movements were similarly minor. Physical growth may also drive changes in arch orientation, as ML arch movement decreased in preterm pigs, which might reflect an elongation of their anatomy in the AP direction and subsequent shortening of their anatomy in the ML direction. As infants, especially those born prematurely, possess a compressed oropharyngeal anatomy, we would expect to see greater changes in preterm infants than term infants, which is reflected by our data. Overall, our data suggest that studying the oropharynx as a two, rather than 3D space may limit our understanding of the function of the structures that power swallowing. Further, the differences between term and preterm infant anatomy may play a role in the decreased contractility in the pharynx in premature infant humans (Prabhakar et al. 2019).

### Preterm infants experience maximal arch constriction earlier than term infants

Unlike total excursion measurements, data collected on the timing of maximal palatopharyngeal arch constriction is not affected by inconsistency in marker placement. Thus, we can assume that constriction timing signals were both precise and accurate. While decreased soft palate excursion in preterm infants suggests temporal disorganization of the five muscles that control the soft palate, earlier palatopharyngeal arch constriction may suggest a more general disorganization of the different parts of anatomy involved in the swallow. Early maximal arch constriction may result in a number of negative consequences. For example, early arch constriction could suggest that the epiglottis returns to its resting position earlier in the swallow, which would not allow milk to drain completely from the valleculae and into the esophagus, resulting in decreased swallow safety. Coordination of arch constriction and bolus transport is thought to be crucial in ensuring that enough pressure is generated to propel the bolus into the esophagus (McConnel 1988; Pongpipatpaiboon et al. 2017), and the timing we observed in preterm infants may be reflective of poor coordination between the two. This may explain why studies using pharyngeal manometry show differences in contractility and associated pressure generation in preterm and term infants (Prabhakar et al. 2019). To explore this possibility, future studies could integrate pharyngeal manometry with 3D tracking of palatopharyngeal arch kinematics. If constriction of the palatopharyngeal arch occurs too early in the swallow, then the bolus may not have traveled far enough down the oropharynx, and thus the positive pressure generated by arch constriction may not be sufficient to move the entire bolus into the esophagus.

### Conclusions

This study is the first to extensively analyze the 3D kinematics of the palatopharyngeal arch in order to characterize its role in swallowing. Our results support recent work that implies that preterm birth impacts the coordination between structures more so than the movements of isolated structures (Catchpole et al. 2020; Mayerl et al. 2020b). Further, by studying arch and soft palate function in a pathophysiological population (Barlow 2009; Bryant-Waugh et al. 2010; Flamand et al. 2012; Pitcher et al. 2012), we have been able to begin to understand their functions in swallowing. First, soft palate movements, which are essential for both maintaining a seal to propel the bolus as well as protect the nasopharynx during a swallow (Crompton et al. 1997; Pongpipatpaiboon et al. 2017), are decreased in preterm infants. This is likely a result of a reliance of soft palate movements on not only constriction of the palatopharyngeal muscles, but also on coordinated activation of the tensor veli palatini, levator veli palatini, palatoglossus, and the uvula. Second, increased delays in maximal constriction of the palatopharyngeal arch may play a role in maintaining airway protection during swallowing by maintaining a retracted epiglottis for the duration of the swallow (Crompton et al. 2008; Mayerl et al. 2020c). These possibilities could be tested through electromyographic recordings of the muscles involved, or by measuring how the movements of different oropharyngeal structures covary with performance measures such as airway protection or bolus size. Additionally, by using a longitudinal study design, we show that arch movements and function change postnatally as animals change their feeding posture and become more anatomically elongated, suggesting that the neural control of swallowing must similarly show some level of postnatal maturation. Together, these results suggest that the timing and coordination of oropharyngeal movements may be more important to feeding performance than the movements of individual structures, and that understanding feeding function requires an integrated perspective.

## Author contributions

C.E.E., R.Z.G., and C.J.M. designed the study and wrote the manuscript. C.E.E. and C.J.M. processed the data and statistical analyses. All authors collected the data, edited the manuscript, and approved the manuscript for publication. 

## Supplementary Material

obaa028_Supplementary_Data
